# HER2 status is positively associated with vessel invasion of colorectal cancer: a retrospective large cohort study

**DOI:** 10.1007/s00384-022-04243-2

**Published:** 2022-08-25

**Authors:** Mingdian Wang, Xiang Wang, Yiwei Li, Qingguo Li, Sanjun Cai, Xinxiang Li, Maoguang Ma

**Affiliations:** 1 Department of Colorectal Surgery, Fudan University Shanghai Cancer Center, Shanghai, China; 2 Department of Oncology, Shanghai Medical College, Fudan University, Shanghai, China; 3 Department of Pathology, Collaborative Innovation Center for Cancer Medicine, Sun Yat-Sen University Cancer Center, Guangzhou, Guangdong Province, China; 4 Department of General Surgery, the Second People’s Hospital of Hefei, Hefei Hospital Anhui Medical University, Hefei, Anhui Province, China

**Keywords:** Colorectal cancer, HER2, Immunohistochemistry, Vessel invasion

## Abstract

**Purpose:**

HER2-positive colorectal cancer was drawn increasing attention in recent years. Accumulating evidence showed HER2-positive metastatic colorectal cancer could benefit from HER2-targeted therapy. While HER2 expression and the relationship between HER2 status and clinicopathological characteristics of overall colorectal cancer remains largely unknown. The aim of this study was to evaluate HER2 expression in colorectal cancer and compare the clinicopathological features between HER2-positive and HER2-negative colorectal cancer.

**Methods:**

We retrospectively analyzed 3910 primary colorectal cancer patients treated in our institution from January 2016 to December 2019. Medical records and pathology reports after surgery were collected to provide information about HER2 status and other clinicopathological characteristics.

**Results:**

We identified 3347 HER2-negative and 79 HER2-positive colorectal cancer patients in our cohort. The chi-square test showed that vessel invasion was significantly more common in HER2-positive colorectal cancer patients. Crude analysis showed HER2 positive was associated with vessel invasion in colorectal cancer [OR and 95% CI 0.534 (0.341, 0.835), *p* = 0.006]. After adjusting for N stage, a significant association was still observed between HER2 status and vessel invasion in colorectal cancer [OR and 95% CI 0.550 (0.322, 0.941), *p* = 0.029]. Survival analysis showed that there was no significant difference in 3-year overall survival rate between HER2 positive and HER2 negative group (*p* = 0.603).

**Conclusion:**

Our findings indicate that the rate of HER2 positivity in colorectal cancer was relatively low, and HER2 status was strongly associated with vessel invasion while having no significant influence on the 3-year overall survival rate in colorectal cancer patients.

## Introduction

Human epidermal growth factor receptor 2 (HER2) is one of the members of the EGFR family of receptor tyrosine kinases [1]. Former studies had proved that HER2 overexpression is closely related to tumorigenesis and progression [2–4]. In addition, HER2 is overexpressed in about 30% of breast cancer and 20% of advanced gastric cancer [5–8]. Based on the evidence from previous clinical trials, HER2 had been well-established as a therapeutic target in breast cancer and gastric cancer [9–13].

In recent years, many studies revealed that HER2 is a promising target in HER2-amplified metastatic colorectal cancer (CRC) [14–16]. Moreover, HER2 could be a negative biomarker for EGFR-targeted treatments in CRC [17]. However, the most current research was focused on HER2 positive metastatic CRC (mCRC), and the clinical and pathological features of overall HER2-positive CRC remain largely unknown.

In this retrospective study, we aimed to compare the clinical and pathological features between HER2 negative and HER2 positive CRC and provide meaningful information about HER2-positive CRC.

## Methods

### Patients

This study was approved by the ethics committee of Fudan University Shanghai Cancer Center (No.2206255-Exp1). Patients newly diagnosed with CRC between January 2016 and December 2019 were retrospectively analyzed in this study. The inclusion criteria were as follows: (1) primary CRC with radical resection. (2) primary CRC with complete records of clinical and pathological data, including sex, age, T stage, N stage, distant metastasis, vessel invasion, perineural invasion, tumor differentiation, and HER2 status. (3) primary CRC without neoadjuvant treatment. (4) primary CRC without other severe diseases, especially cancer.

Clinical and pathological data were collected from the department of information of our center, including age, sex, TNM stage, vessel invasion, perineural invasion, tumor differentiation, and HER2 status. These patients were followed up by telephone or by visiting the hospital. The follow-up interval was 3 months for the first 2 years after surgery and 6 months for the third year after surgery. The overall survival (OS) was defined as the time from surgery to death or the last follow-up.

### HER2 testing

HER2 status was measured by immunohistochemistry, which was evaluated according to the HERACLES criteria (IHC score 0: no staining or staining in < 10% of cells; IHC score 1 + : faint staining or moderate staining < 50% of cells or intense staining ≤ 10% cells; IHC score 2 + : moderate staining in ≥ 50% of cells; IHC score 3 + : intense staining in ≥ 50% of cells. In situ hybridization (ISH) was mandatory when IHC score 2 + . HER2 positivity, defined as IHC score 3 + and ISH HER2: CEP17 ratio higher than two in more than 50% of cells).

Only IHC score 0–1 + was considered HER2 negative, and IHC score 3 + was considered HER2 positive in this study due to ISH of CRC was not routinely performed in our institute.

### Statistical analysis

SPSS version 23.0 was recruited to conduct all statistical analyses. Student’s *t*-test was used to compare differences in continuous variables between HER2 negative and HER2 positive groups. The chi-square test or Fisher’s exact test was used to compare categorical variables between HER2 negative and HER2 positive groups. Univariate and multivariate logistic regression models were applied to assess the influence of HER2 status on clinical and pathological characteristics by calculating the odd ratios and their corresponding 95% confidence intervals (CIs). The cumulative survival probabilities of patients were estimated using the Kaplan–Meier method and compared with the log-rank test. *P* < 0.05 was considered to be statistically significant.

## Results

### Relationship between HER2 status and clinicopathologicl factors

**Fig. 1:**
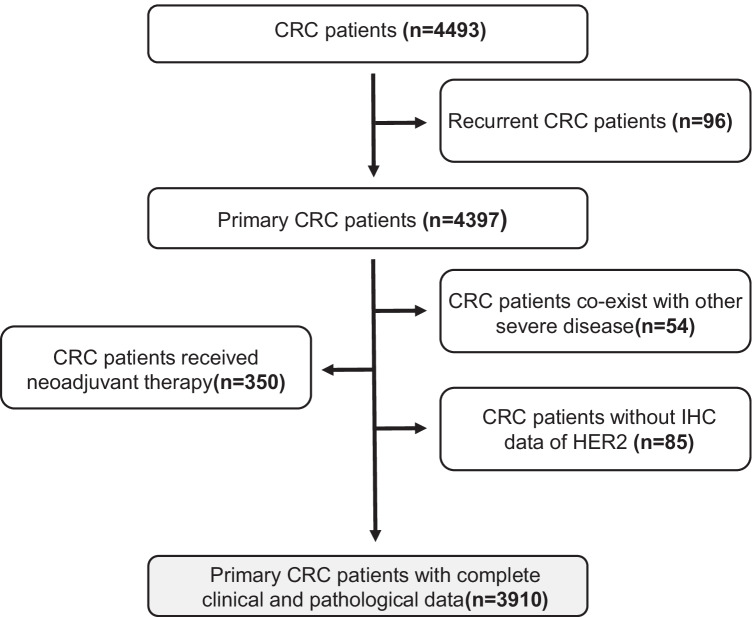
Flow diagram of the patient selection process-01

We collected 4493 CRC patients treated in our department from January 2016 to December 2019. Among them, 96 were recurrent CRC cases, 404 patients co-exist with other severe diseases or received neoadjuvant treatment (12 with a lung cancer history, 25 with breast cancer history, 17 with a renal cancer history, and 350 received neoadjuvant chemotherapy or radiotherapy), and 85 cases without IHC results of HER2 for unknown reasons. A total of 3910 primary CRC cases with complete clinical and pathological data were enrolled in this study (Fig. 1).

The overall HER2 status in this cohort tested by IHC was shown in Table 1. According to the HER2 testing criteria described above, 3347 cases were regarded as HER2 negative CRC and 79 as HER2 positive in total.

**Table 1:** Demographics and clinical characteristics of patients in this study

**Characteristics**	**IHC ( −)** ***N*** **(%)/mean ± sd**	**IHC (1 +)** ***N*** **(%)/mean ± sd**	**IHC (2 +)** ***N*** **(%)/mean ± sd**	**IHC (3 +)** ***N*** **(%)/mean ± sd**
**Age (year)**	60.35 ± 11.89	60.51 ± 11.61	60.24 ± 11.72	58.29 ± 10.39
**Sex**				
Male Female	1480 (60) 945 (40)	599 (65) 323 (35)	318 (65.7) 166 (34.3)	43 (54.4) 36 (45.6)
**T stage**				
T1 T2 T3 T4	133 (5.5) 347 (14.3) 1566 (64.6) 379 (15.6)	34 (3.7) 128 (13.9) 631 (68.4) 129 (14)	22 (4.5) 67 (13.8) 335 (69.2) 60 (12.4)	7 (8.9) 9 (11.4) 52 (65.8) 11 (13.9)
**N stage**				
N0 N1 N2	1318 (54.4) 670 (27.6) 437 (18)	524 (56.8) 254 (27.6) 144 (15.6)	267 (55.2) 139 (28.7) 78 (16.1)	36 (45.6) 27 (34.2) 16 (20.3)
**Distant metastasis**				
Yes No	2084 (85.9) 341 (14.1)	765 (83) 157 (17)	390 (80.6) 94 (19.4)	69 (87.3) 10 (12.7)
**TNM stage**				
I II III IV	360 (14.9) 856 (35.3) 869 (35.8) 340 (14)	127 (13.8) 351 (38.1) 289 (31.3) 155 (16.8)	61 (12.6) 181 (37.4) 148 (30.6) 94 (19.4)	10 (12.7) 23 (29.1) 36 (45.6) 10 (12.7)
**Vessel invasion**				
Yes No	1489 (61.4) 936 (38.6)	595 (64.5) 327 (35.5)	310 (64) 174 (36)	37 (46.8) 42 (53.2)
**Perineural invasion**				
Yes No	1616 (66.6) 809 (33.4)	615 (66.7) 307 (33.3)	305 (63) 179 (37)	47 (59.5) 32 (40.5)
**Tumor differentiation**				
High Poor-moderate	2293 (94.6) 132 (5.4)	877 (95.1) 45 (4.9)	455 (94) 29 (6)	76 (96.2) 3 (3.8)
**Total**	2425 (62)	922 (23.6)	484 (12.4)	79 (2)

Then we divided these patients into two groups by HER2 status and analyzed the association between clinicopathological characteristics and HER2 status (Table 2). The average age was 58.29 and 60.43 years old in the HER2 positive and HER2 negative groups, respectively. There was no significant difference in age between these two groups (*p* = 0.113). Moreover, chi-square analysis showed that there were no significant differences between sex, T stage, N stage, distant metastasis, TNM stage perineural invasion, and tumor differentiation with HER2 status. However, significant differences were found between vessel invasion and HER2 positive and HER2 negative groups (*p* = 0.005).

**Table 2:** Number of patients in HER2 positive and HER2 negative groups, respectively

	**HER2 positive** ***N*** **(%)/mean ± sd**	**HER2 negative** ***N*** **(%)/mean ± sd**	**Statistic**	***P***
**Age (year)**	58.29 ± 10.391	60.43 ± 11.89	− 1.587	0.113
**Sex**				
Male Female	43 (54.4) 36 (45.6)	2079 (62.1) 1268 (37.9)	1.933	0.164
**T stage**				
T1 T2 T3 T4	7 (8.9) 9 (11.4) 52 (65.8) 11 (13.9)	167 (5) 475 (14.2) 2197 (65.6) 508 (15.2)	2.717	0.43
**N stage**				
N0 N1 N2	36 (45.6) 27 (34.2) 16 (20.3)	1842 (55) 924 (27.6) 581 (17.4)	2.833	0.243
**Distant metastasis**				
Yes No	10 (12.7) 69 (87.3)	498 (14.9) 2849 (85.1)	0.301	0.583
**TNM stage**				
I II III IV	10 (12.7) 23 (29.1) 36 (45.6) 10 (12.7)	487 (14.6) 1207 (36) 1158 (34.6) 495 (14.8)	4.132	0.248
**Vessel invasion**				
Yes No	42 (53.2) 37 (46.8)	1263 (37.7) 2084 (62.3)	7.791	0.005
**Perineural invasion**				
Yes No	32 (40.5) 47 (59.5)	1116 (33.3) 2230 (66.7)	1.772	0.183
**Tumor differentiation**				
High Poor–moderate	3 (3.8) 76 (96.2)	177 (5.3) 3170 (94.7)	0.798	0.396

Logistic regression univariate and multivariate analyses were used to analyze the associations between HER2 status and other clinical and pathological characteristics. On crude analysis, HER2-positive CRC patients were more likely to with vessel invasion, OR and 95% CI 0.534 (0.341, 0.835), *p* = 0.006. After adjusting for N stage, a similar association was seen, OR and 95% CI 0.550 (0.322, 0.941), *p* = 0.029. While sex, T stage, N stage, distant metastasis, TNM stage, perineural invasion, and tumor differentiation were not showed significant association with HER2 status (Table 3).

**Table 3:** Odds ratios (OR) (with 95% CI) of HER2 positive relative to HER2 negative by different characteristics of CRC patients

		**Crude**	**Adjusted**
		OR (95% CI) *P*	OR (95% CI) *P*
**Sex**	Male Female	Ref. 0.729 (0.465, 1.141) 0.166	\
**T stage**	T1–T2 T3–T4	Ref. 1.072 (0.615, 1.868) 0.806	\
**N stage**	N0 N1–N2	Ref. 0.684 (0.437, 1.071) 0.097	Ref. 0.946 (0.553, 1.619) 0.839
**Distant metastasis**	Yes No	Ref. 1.206 (0.617, 2.357) 0.584	\
**TNM stage**	I-II III-IV	Ref. 0.700 (0.445, 1.100) 0.122	\
**Vessel invasion**	Yes No	Ref. 0.534 (0.341, 0.835) 0.006	Ref. 0.550 (0.322, 0.941) 0.029
**Perineural invasion**	Yes No	Ref. 0.735 (0.466, 1.158) 0.184	\
**Tumor differentiation**	High Poor–moderate	Ref. 1.415 (0.442, 4.528) 0.559	\

### Prognostic value of HER2 status in CRC patients

**Fig. 2:**
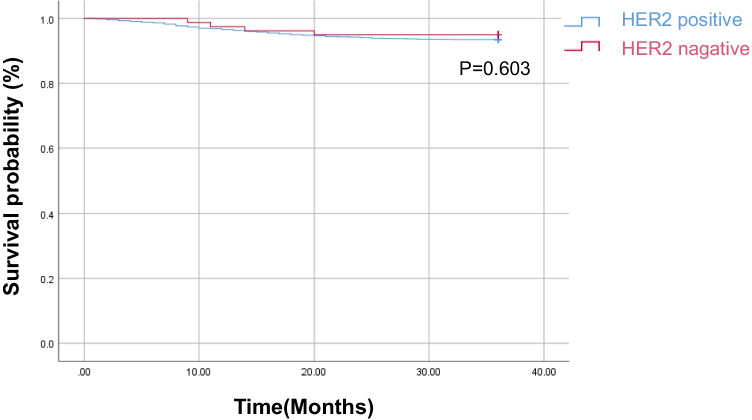
OS curve of colorectal cancer patients based on HER2 status

Furthermore, we analyzed the relationship between HER2 status and 3-year overall survival (OS) in CRC patients. A total of 3257 patients in the HER2 negative group and 79 in the HER2 positive group were followed up, and the follow-up rate was 97.7%. The results showed that there was no significant difference in OS rate between the HER2 positive and HER2 negative groups (*p* = 0.603, Fig. 2).

## Discussion

The diagnostic criterion for HER2 positivity in CRC has not reached an agreement worldwide. The rate of HER2 positivity in CRC varied widely in different researches due to inconsistent diagnostic criteria [18].

In this study, we adopted the HERACLES criteria, which were proposed by Valtorta and his colleagues [19]. They established an archival test cohort which was conducted on formalin-fixed paraffin-embedded archival samples by three pathologists and concluded an agreement by collegial review and discussion, thus formulating a diagnostic algorithm for HER2 positivity in CRC, referred to as HERACLES Diagnostic Criteria. Then, the clinical validation cohort was established on KRAS 12/13 wild-type mCRC patients to validate the accuracy of this criteria.

HER2 expression in CRC can also be detected by molecular techniques except for IHC and ISH. Shimada Y et al. proved that comprehensive genomic sequencing (CGS) has the same utility as IHC and ISH for detecting HER2-positive CRC patients [20]. Nakamura Y et al. demonstrated that circulating tumor DNA (ctDNA) is an efficient marker for predicting *HER2*-amplified mCRC in clinical practice [21].

Some clinical trials had acquired promising results regarding HER2-targeted therapy in mCRC. The most well-known studies were HERACLES and MyPathway [22, 23]. HERACLES study preliminary proved that trastuzumab combined with lapatinib was effective in treating *KRAS* exon 2 wild type, HER2 positive mCRC (response rate was 30%, 4% for complete response, and 26% for partial response). MyPathway study showed that pertuzumab plus trastuzumab was well tolerated and effective in treatment-refractory HER2-amplified mCRC (response rate was 32%, 2% for complete response, and 30% for partial response). Grelly et al. summarized 9 completed or ongoing clinical trials concerning HER2-targeted therapy in mCRC [24]. Though HER2-targeted therapy showed promising results in mCRC patients, the prognostic role of HER2 in CRC remains unclear, further large cohort studies with stratified and long-term follow-up dates were required to elucidate this point.

Several previous studies also analyzed the association between HER2 status and pathological characteristics in CRC patients. Zhang et al. reported that HER2 status was significantly associated with T stage and TNM stage in CRC, but not associated with lymphovascular invasion [25]. Seo et al. reported that HER2 amplification was not associated with any pathological variables except tumor location in the rectum [26].

In conclusion, our study demonstrated that the rate of HER2 positive in CRC was relatively low. Moreover, HER2-positive CRC patients were more likely with vessel invasion than HER2-negative CRC patients. However, while had no significant influence on 3-year overall survival in CRC patients.

## Data Availability

The datasets generated during and/or analyzed during the current study are available from the corresponding author on reasonable request.
